# Development of the Canadian Women-Centred HIV Care Model Using the Knowledge-to-Action Framework

**DOI:** 10.1177/2325958221995612

**Published:** 2021-04-13

**Authors:** Mona Loutfy, Wangari Tharao, Mina Kazemi, Carmen H. Logie, Angela Underhill, Nadia O’Brien, Neora Pick, Mary Kestler, Mark H.Yudin, Jesleen Rana, Jay MacGillivray, V. Logan Kennedy, Denise Jaworsky, Adriana Carvalhal, Tracey Conway, Kath Webster, Melanie Lee, Shaz Islam, Valerie Nicholson, Mary Ndung’u, Karène Proulx-Boucher, Allison Carter, Rebecca Gormley, Manjulaa Narasimhan, Alice Welbourn, Alexandra de Pokomandy, Angela Kaida, on behalf of the CHIWOS Research Team

**Affiliations:** 1 Women’s College Research Institute, Women’s College Hospital, Toronto, Canada; 2 Faculty of Medicine, University of Toronto, Toronto, Canada; 3 Women’s Health in Women’s Hands Community Health Centre, Toronto, Canada; 4 Factor-Inwentash Faculty of Social Work, University of Toronto, Toronto, Canada; 5 Department of Family Medicine, McGill University, Montreal, Canada; 6 Oak Tree Clinic, BC Women’s Hospital, Vancouver, Canada; 7 Department of Obstetrics and Gynecology, St. Michael’s Hospital, Toronto, Canada; 8 Northern Medical Program, University of Northern British Columbia, Prince George, Canada; 9 Department of Psychiatry, Queen’s University, Kingston, Canada; 10 Faculty of Health Sciences, Simon Fraser University, Burnaby, Canada; 11 Alliance for South Asian AIDS Prevention, Toronto, Canada; 12 Chronic Viral Illness Service, McGill University Health Centre, Montreal, Canada; 13 Kirby Institute, UNSW Sydney, Australia; 14 BC Centre for Excellence in HIV/AIDS, Vancouver, Canada; 15 Department of Sexual and Reproductive Health Research, World Health Organization, Geneva, Switzerland; 16 Salamander Trust, United Kingdom lead coordinator of the Global Values and Preferences Survey.

**Keywords:** HIV, Women, model of care, women-centred HIV care, implementation science

## Abstract

In Canada, women make up 25% of the prevalent HIV cases and represent an important population of those living with HIV, as a high proportion are racialized and systemically marginalized; furthermore, many have unmet healthcare needs. Using the knowledge-to-action framework as an implementation science methodology, we developed the “Women-Centred HIV Care” (WCHC) Model to address the needs of women living with HIV. The WCHC Model is depicted in the shape of a house with trauma- and violence-aware care as the “foundation”. Person-centred care with attention with attention to social determinants of health and family make up the “first” floor. Women’s health (including sexual and reproductive health and rights) and mental and addiction health care are integrated with HIV care, forming the “second” floor. Peer support, leadership, and capacity building make up the “roof”. To address the priorities of women living with HIV in all their diversity and across their life course, the WCHC Model should be flexible in its delivery (e.g., single provider, interdisciplinary clinic or multiple providers) and implementation settings (e.g., urban, rural).

**1. What do we already know about this topic?**

Representing 25% of people living with HIV in Canada, women living with HIV face social and health inequities and gaps in care related to social determinants of health, mental, sexual, reproductive, and women’s health.

**2. How does your research contribute to the field?**

This work presents the implementation science methods used to develop a comprehensive model of care for women living with HIV and asserts that women’s health with consideration of sexual and reproductive health and rights, and mental and addiction health should be integrated with HIV care in a trauma-aware, person-centred care model with peer support as an integral component.

**3. What are your research’s implications towards theory, practice, or policy?**

This research results in a well-needed model of care that can be implemented in various settings to address the unique needs and priorities of women living with HIV in all their diversity and across their life course.

## Introduction

Worldwide, women make up over 50% of people living with HIV.^1^ In Canada, the prevalence of HIV in women has steadily risen over the past 2 decades and plateaued at about 25%.^2,3^ However, the demographics of women living with HIV in Canada are unique. While 42% of men acquiring HIV in Canada are white, only 14% of women are, with the majority of new cases disproportionately affecting Indigenous and African, Caribbean, and Black women.^4^ Also, only 13% of men report injection drug use as their mode of acquisition compared to 28% of women.^2^ In addition to biological factors, social and structural factors such as poverty, racism, gender, and power inequities increase women’s susceptibility to HIV acquisition.^5^ Along with these social and structural factors, the intersection of sexual and reproductive health rights and concerns, such as unmet contraception needs, exacerbate HIV hardships for women.^6^ Furthermore, studies across the global North have shown that women living with HIV experience higher rates of depression,^7^ HIV-related stigma^8^ and poorer outcomes along the HIV care cascade compared to men.^9-12^ In Canada, these inequities contribute to a lower life expectancy for women compared to men living with HIV.^13^

The comprehensive care needs of women living with HIV in Canada have not been thoroughly addressed due to the historic focus of the HIV response on men who have sex with men,^14^ and the underrepresentation of women in decision-making roles.^15^ Consequently, gender-specific care gaps exist for women living with HIV throughout their lives, including: sexual and mental health support, contraceptive options, cervical and breast cancer screening and treatment, counselling on aging, menopause, co-infections and comorbidities, fertility services, pregnancy planning and safe delivery, postpartum care, intimate partner violence screening and interventions, and services that address social determinants of health.^16^

The significant gap in clinical care, practice, and policy for girls and women living with HIV is not unique to Canada. This was highlighted in the Salamander Trust’s Global Values and Preferences survey (GVPS) commissioned by the World Health Organization (WHO) to inform the consolidated guideline on sexual and reproductive health and rights (SRHR) of women living with HIV.^14,17^ Designed and conducted by and for women living with HIV in all their diversity, the GVPS includes feedback from 945 women in 94 countries. Survey respondents expressed the desire for “a holistic, woman-centred con(tra)ception-to-old-age approach to sexual and reproductive health care, with a comprehensive package of age- and stage-appropriate services”.^17(p5)^ A key finding was high levels of violence—of the 58% of women who responded to the optional online section on violence (n = 480), 89% revealed that they either feared or experienced violence. Of the 59% of women who responded to the online optional section on mental health, 74% reported experiences of depression, 71% shame and 70% feelings of rejection after or because of their HIV diagnosis.^17,18^ Based on the principles of human rights and gender equality, the WHO guideline on SRHR of women living with HIV incorporated the findings from the GVPS and issued 51 WHO recommendations and 22 WHO good practice statements for policy and programming to advance the SRHR of women living with HIV.^14^

Using the knowledge-to-action framework as an implementation science methodology,^19-21^ we developed a comprehensive, holistic and flexible model of care for women living with HIV in Canada, the Women-Centred HIV Care (WCHC) Model, which is in line with the GVPS and the WHO guideline on SRHR of women living with HIV. The Canadian knowledge-to-action framework was developed specifically to identify the health issues in a population, determine the gaps in addressing this health issue, and to develop interventions or solutions to address the gaps. We, therefore, found it ideal for the development of our model of care. The aim of this paper is to describe the WCHC Model and the processes used in its development. Drawing on stakeholder feedback, we also present model implementation considerations and barriers. While the WCHC Model has been developed for the Canadian context, it may be transferable for the care of women living with HIV in other countries where there is an unmet need.

## Methods

The knowledge-to-action framework, the basis of our care model development methods, was developed in Canada by Dr. Ian Graham and colleagues and is a required component of federally funded grants.^19^ It is a conceptual framework, often referred to as an implementation science methodology, intended to guide the creation and synthesis of knowledge which is then to be converted into usable practice tools that impact health outcomes. It consists of 3 knowledge creation phases and 7 action cycle phases. It is meant to be iterative and dynamic and often the phases occur in tandem or out of the initially conceptualized order.^19^

**Figure 1.**
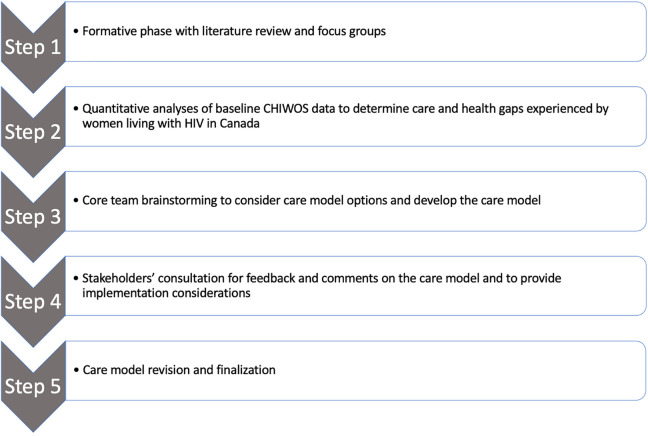
Five care model development steps.

We used the 3 knowledge creation phases: knowledge inquiry, synthesis, and product development and 3 of the 7 action cycle phases: “identifying the problem” and “reviewing and selecting the knowledge useful for action”, “adapting the knowledge to the local context” (the Canadian context), and “assessing barriers to knowledge use.” We took these 6 knowledge-to-action phases and packaged them into 5 care model development steps: 1) a formative step with a literature review and focus groups, 2) a quantitative analyses step to determine care gaps, 3) a brainstorming step with the core team to consider care model options and develop the care model, 4) a stakeholders’ consultation step which not only contributed to model revisions but also the consideration of operational barriers, and finally 5) a care model revision and finalization step. Our 5 care model development steps are outlined in **Figure 1**.

### Step 1. Formative Phase

To understand the literature on and define women-centred care in the field of HIV, we started with a formative phase which included 1) a literature review and 2) focus groups with women living with HIV. The literature review was carried out using scoping review methodology^22,23^ and the objective was to explore the concept of women-specific HIV/AIDS services to identify and define what key elements underlie this approach to care. The review methods are published elsewhere.^23^ After the literature review, the term used by our team was changed from “women-specific HIV/AIDS services” to “women-centred HIV care” in order to be inclusive of all women and their families and to include overall care rather than individual services. We then realized that the important next step was to ask women living with HIV, themselves, what women-centred HIV care meant to them. This was done by carrying out 11 focus groups in 2012 with 77 women living with HIV in 3 Canadian provinces (British Columbia [BC], Ontario and Quebec). The methods have been previously published.^16,24,25^

### Step 2. Quantitative Analyses

Using community-based research principles^26^ and prioritizing the requirements of the “Greater Involvement of People living with HIV/AIDS”,^27^ we partnered with women living with HIV along with clinicians and academics to develop our 2136-item questionnaire used in the Canadian HIV Women’s Sexual and Reproductive Health Cohort Study (CHIWOS).^28^ Women living with HIV were trained to be research assistants (RAs) and they, along with 3 allied RAs, obtained participants’ consent and administered the survey using a web-based platform.^29^ From August 2013 to May 2015, 1422 women living with HIV in BC, Ontario, and Quebec were enrolled and completed the baseline questionnaire.^30^ Follow-up interviews were conducted for 2 more time points at 18-month intervals. For the purpose of developing the WCHC Model, findings exclusively from the baseline visit were used due to availability. Guided by the women living with HIV on the team, we prioritized analyses and publications of the issues deemed most pressing by the RAs and participants. It was the findings from these original analyses, papers, and conference abstracts that directed the development of the care model.

### Step 3. Core Team Brainstorming and Care Model Development

The findings from the literature review, focus groups, and the quantitative baseline visit analyses were presented to the core research team in 2015 and an intensive discussion was held to consider the components and structure of the WCHC Model. As “knowledge inquiry” was done in step 1 and 2, this third step encompassed the 2 final phases of the knowledge-to-action framework’s knowledge creation: “synthesis” and “product development” (the product being the WCHC Model).

### Step 4. Stakeholders’ Consultations

Once the first draft of the WCHC Model was developed, the next step was to obtain feedback from key external partnering stakeholders. The model was sequentially circulated, and feedback was obtained from the Ontario CHIWOS RAs, the CHIWOS WCHC Model Working Group and a large group of Ontario clinics and community-based organizations (CBOs) that provide the majority of care to women living with HIV in the province. Next, the larger Toronto Infectious Diseases (ID) and Internal Medicine community and the CHIWOS RAs from BC and Quebec reviewed the model and provided feedback. For the final review in Canada, focus groups and one-on-one interviews were held with 76 women living with HIV (59 from Ontario and 17 from BC) and 7 CBO service providers. Finally, international feedback was obtained from 2 key international stakeholders (MN, AW) and the model was presented at the 8th International Workshop on HIV and Women^31^ to allow further feedback on international applicability. Stakeholders’ feedback was captured by computer note-taking or audio recordings and transcription.

### Step 5. Care Model Revision and Finalization

Finally, the WCHC Model was revised to incorporate the wider team and stakeholder feedback, and implementation barriers and other considerations that were noted. A final model of care was designed with the creative and artistic direction from the The Public Studio.

### Ethical Approval and Informed Consent

Primary ethics approval was obtained from Women’s College Hospital (Ontario), Simon Fraser University (BC), University of British Columbia/Providence Health (BC), and McGill University Health Centre (Quebec) from their respective Research Ethics Boards. Written informed consent to participate in the study was obtained from all participants.

## Results

Here we outline the key outputs from each step of developing the WCHC Model. Steps 3 and 5 have been combined into the presentation of the final WCHC Model.

### Formative Phase—Defining WCHC

The literature review revealed that in order to be effective, women-specific services must adopt approaches to care that recognize women’s unique health and social priorities and the intersections between women’s experiences (e.g., the social, political, economic context of their lives) and their health.^23^ We determined that “women-specific HIV services” is a complex, multi-dimensional concept for which a consistent definition did not exist.^23^ The concept was refined into 12 dimensions which were presented in the form of a flower.^23^ Overall, one major theme emerged: recognizing and responding to women’s unique SRHR and social care priorities must be at the core of HIV programming for women.

From the focus groups findings, women living with HIV envisioned a women-centred HIV care approach as one that builds on basic care competencies (i.e. the care provider has sufficient HIV knowledge to provide quality HIV care), is grounded in person-centred care principles,^32^ and acknowledges both women’s health, including their SRHR care, and their HIV care priorities. In addition, the women indicated that care must acknowledge and respond to structural barriers that limit women’s care access such as violence, poverty, motherhood, HIV-related stigma, and challenges to safe disclosure.^16^ Notably, women saw WCHC as care that fosters peer support and peer leadership in its design and delivery, and honours the diversity of women’s experiences as it relates to the intersection of their race, ethnicity, gender, sexuality, class, citizenship status, ability, primary language, religion, family makeup, and more. They saw peer support and leadership as a way to overcome women’s isolation and prioritize women’s ownership over the decisions that affect their lives.^16^

The completion of the literature review and the focus groups led to our definition of WCHC:

Care that supports women living with HIV to achieve the best health and wellbeing as defined by them. This type of care recognizes, respects, and addresses women’s unique health and social concerns, and recognizes that they are connected. Because this care is driven by women’s diverse experiences, it is flexible and takes different needs into consideration.^30(p3)^

### Quantitative Analyses—Determining the Care Gaps

In the quantitative study phase, an extensive questionnaire with 9 sections was interviewer-administered to 1422 women, who were ≥16 years of age, living with HIV in BC, Ontario and Quebec and was inclusive of cis and trans women, as well as two-spirit, gender non-binary and gender-diverse people who identified, in some ways and/or at some times, as women. Two-spirit is a cultural term used by many Indigenous people to describe people who have a gender and/or spiritual identity that is outside of the colonial, binary gender categories of masculinity and femininity; historically, some two-spirit individuals were believed to have been healers and clairvoyants in their communities, or held other roles that were not limited by gender.^33,34^ People who identify as gender non-binary also identify with a gender identity outside of the binary of woman or man; academically, these individuals and many others believe that the binary nature of gender was invented and enforced by the Eurocentric colonizers for the self-benefit of cis men at the time. Non-binary folks may resonate with masculine or feminine identities, both, or neither.^35^ We recognize that our open interpretation of women in some ways facilitated inclusivity, and in other ways limited our recruitment and findings; a smaller team is currently exploring the applicability (and necessary changes) of the model to better serve more gender diverse groups. Throughout the paper, participants are referred to as “she/her” and this is limited by our recruitment strategy that did not ask about preferred pronouns; we recommend more inclusive labelling in the future. The sociodemographic and clinical variables of the participating women are presented in **Table 1**.^30^

**Table 1.** Sociodemographic and Clinical Characteristics of Participating Women Overall and by Province.

Demographic characteristics	N with	Total	British Columbia	Ontario	Quebec	p-value
	responses	*N = 1422*	*N = 356*	*N = 713*	*N = 353*	
**Median Age (IQR)**	1422	43 (36–51)	44 (37–51)	41 (34–49)	46 (38–53)	<0.001
**Gender identity**	1422					
Woman		1359 (96%)	342 (96%)	679 (95%)	338 (96%)	0.804
Trans woman/Two-spirit/Queer/Intersex/Other		63 (4%)	14 (4%)	34 (5%)	15 (4%)	
**Sexual orientation**	1417					
Heterosexual		1237 (87%)	294 (83%)	617 (87%)	326 (92%)	<0.001
LBQQ2S		180 (13%)	61 (17%)	92 (13%)	27 (8%)	
**Ethnicity**	1422					
Indigenous–First Nations, Me´tis or Inuit		318 (22%)	161 (45%)	149 (21%)	8 (2%)	<0.001
African/Caribbean/Black		418 (30%)	28 (8%)	227 (32%)	163 (46%)	
Caucasian/White		585 (41%)	139 (39%)	280 (39%)	165 (47%)	
Other*		103 (7%)	28 (8%)	57 (8%)	17 (5%)	
**Ever incarcerated**	1420	524 (37%)	222 (62%)	205 (29%)	97 (28%)	<0.001
**Injection drug use history**	1396	439 (31%)	225 (63%)	132 (19%)	83 (24%)	<0.001
**Involved in sex work**	1422					<0.001
Yes		82 (6%)	36 (10%)	30 (4%)	16 (5%)	
No		1225 (86%)	300 (84%)	614 (86%)	311 (88%)	
Don’t know/Prefer not to answer		115 (8%)	20 (6%)	69 (10%)	26 (7%)	
**Clinical Characteristics**						
**HCV co-infection**	1415	451 (32%)	201 (56%)	147 (21%)	103 (29%)	<0.001
**HBV co-infection**	1405	119 (8%)	48 (13%)	35 (5%)	36 (10%)	<0.001
**Median years living with HIV (IQR)**	1374	11 (6–17)	12 (7–18)	10 (5–15)	13 (8–18)	<0.001
**Received HIV medical care in last year**	1420	1330 (94%)	350 (98%)	641 (90%)	339 (96%)	<0.001
**Currently taking ART**	1415	1175 (83%)	318 (89%)	534 (75%)	323 (92%)	<0.001
**Undetectable viral load (self-report)#**	1377					<0.001
Undetectable (below 50 c/mL)		1099 (80%)	286 (82%)	503 (74%)	308 (88%)	
Detectable (over 50 c/mL)		204 (15%)	51 (14%)	122 (18%)	31 (9%)	
Don’t know/Prefer not to answer		76 (5%)	13 (4%)	52 (8%)	11 (3%)	
**Most recent CD4 (self-report)**	1382					
<200 cells/mm3		75 (5%)	30 (9%)	22 (3%)	23 (6%)	<0.001
200–500 cells/mm3		386 (28%)	114 (32%)	173 (26%)	99 (28%)	
>500 cells/mm3		698 (51%)	166 (47%)	363 (53%)	169 (48%)	
Don’t know/Prefer not to answer		223 (16%)	42 (12%)	122 (18%)	59 (17%)	

IQR, interquartile range; LBQQ2S, lesbian, bisexual, queer, questioning, or two-spirit; HCV, hepatitis C; HBV, hepatitis B; ART, antiretroviral therapy.

* Other ethnicities included Chinese/Filipino/Japanese/Korean/Latin America/South Asian/Southeast Asian/Arab/West Asian/Multiple ethnicities.

#80% (1097/1377) of the overall cohort self-reported having an undetectable viral load; of the 1175/1415 women on ART, 87% (1025/1175) had an undetectable viral load.

The baseline data collection resulted in 39 peer-reviewed manuscripts and 50 peer-reviewed conference abstracts, over a 4-year period (between September 2015 and July 2019) that informed the development of the model. These findings were used to identify the care and health gaps experienced by the participants. As the results for these manuscripts and conference abstracts are published elsewhere and can be retrieved at www.chiwos.ca, they are not presented in detail. Rather, we present the summary of the key gaps and our interpretation as to how they inform the development of the WCHC Model.

We found that the majority of CHIWOS participants were satisfied with their HIV care (92% were satisfied or very satisfied).^30^ The results across the HIV care cascade revealed that overall, the CHIWOS cohort is approaching the 90-90-90 UNAIDS targets,^4^ with 96% being retained in care, 88% having initiated antiretroviral therapy (ART), 83% being on ART at the time of the survey; and among those on ART, 87% were virally suppressed (<50 copies/mL).^36^ However, there was substantial variability in outcomes by sub-population; women who were younger, Indigenous, recently incarcerated, currently using illicit drugs, and had unstable housing, low income or experienced racial discrimination were much less likely to be on ART or be virologically suppressed.^36,37^

While HIV clinical care outcomes were encouraging, CHIWOS findings revealed other key aspects of the SRHR and wellbeing of women living with HIV that deserve the attention of care providers. Women living with HIV experienced violence at alarmingly high rates. Experiences of violence were nearly universal, with 80% of women reporting they had experienced some form of violence in adulthood.^38^ Specifically, 78% of women reported experiencing physical violence, 93% verbal abuse, 58% controlling violence, and 56% reported experiencing sexual violence.^38^ There was also a high burden of mental health concerns among CHIWOS participants. Of the 1370 women who completed the Center for Epidemiological Studies Depression Scale (CES-D 10), nearly half (49%) reported symptoms consistent with “probable depression” (i.e., CES-D 10 score ≥10).^39^

Specific women’s health concerns were also evident. Current Canadian guidelines recommend annual cervical cancer screening for women living with HIV.^40^ We found that only 69% of eligible CHIWOS participants reported having received a Pap test within the last year, while 18% answered “between 1 and 3 years ago”; and 14% answered that they had their last Pap test more than 3 years ago or *never* had a Pap test.^41^ These trends occurred despite finding that nearly all (95%) women had received HIV medical care in the previous year,^41^ suggesting a missed opportunity for integrating women’s health care within HIV care.

From the stance that SRHR are fundamental to the health and wellbeing of women living with HIV, we found that only 50% of women living with HIV were sexually active^42^ and 50% viewed sexual activity as an important part of their lives.^43^ Significantly, social environments (e.g., stigma, violence, poverty) greatly influenced intimate aspects of women’s lives.^42,43^ Also central to women’s SRHR is pregnancy planning. Of 1,165 women in CHIWOS, 278 (23.9%) reported 492 pregnancies, 60.8% of which were unintended.^44^ This high rate of unintended pregnancy is consistent with limited access and use of hormonal and long-acting reversible contraceptive methods by women living with HIV and inadequate women’s health care.^45^

Finally, while the formative phase focus group participants unanimously indicated that peer support was essential,^24^ formal peer support (i.e. support from other people living with HIV at their clinics and/or other HIV support sites) was only accessed by 36% of CHIWOS survey participants,^46^ suggesting a gap in services. Peer leadership opportunities were identified as important to focus group participants, and yet such occurrences were limited, as only 30% engaged as peer leaders at their HIV clinics.^46^ Women engaged in peer leadership were more likely to be aware of the HIV prevention and other benefits of ART, compared to women who never engaged in peer leadership and women who were unaware of such opportunities (66% and 55%, respectively; p = 0.010).^47^

### The WCHC Model

**Figure 2.**
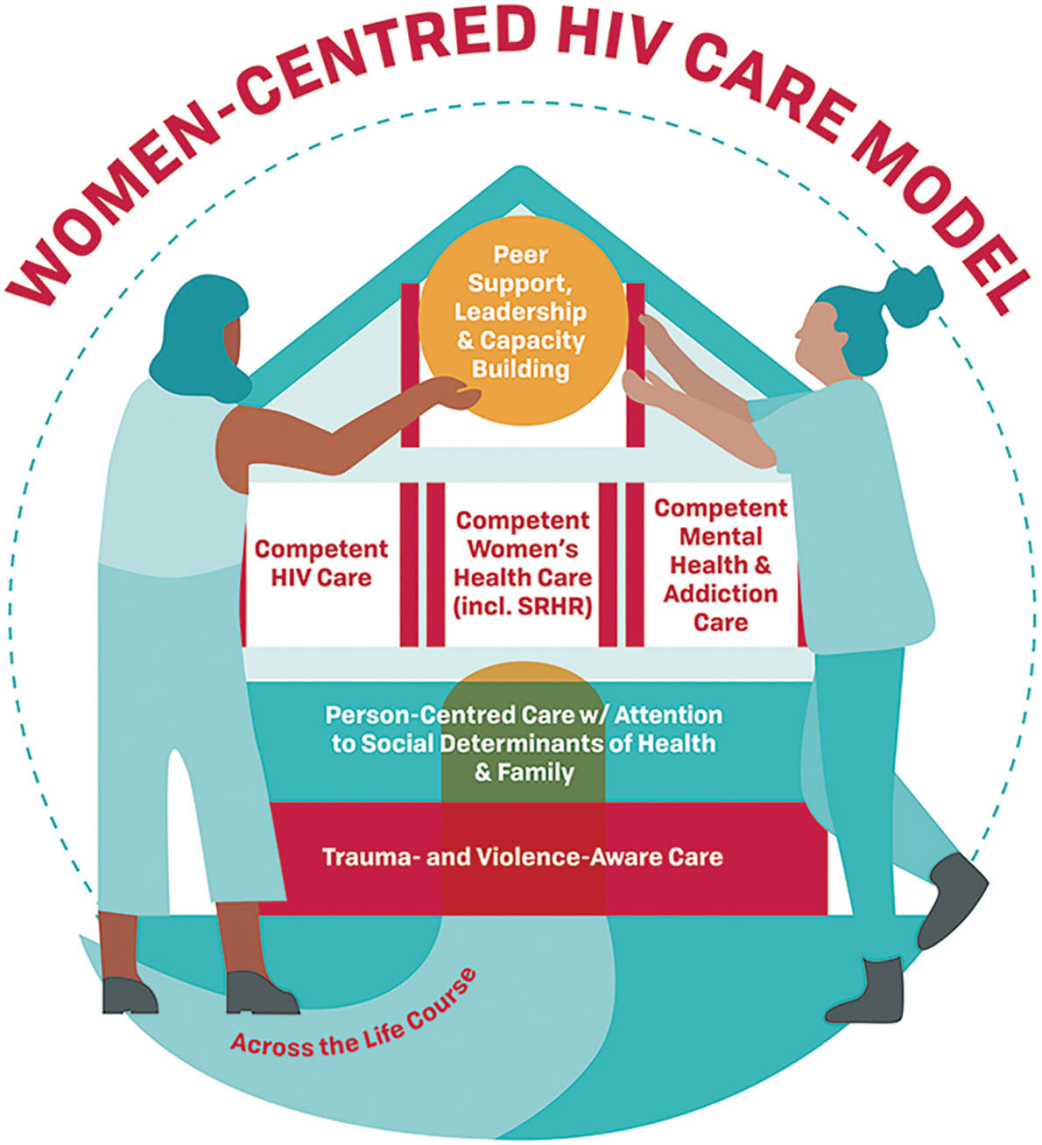
The Women-Centred HIV Care (WCHC) Model. The WCHC model is in the shape of a “house” to represent safety and stability—essential aspects for care delivery. Trauma- and violence- aware care is the “foundation” to acknowledge the alarming high rates of violence, trauma and intersecting oppressions women living with HIV face. Person-centred care with attention to social determinants of health and family make up the “first floor”. For many women, ensuring their family is cared for is essential to their wellbeing as is ensuring adequate housing and livable income as well as other social determinants of health. The “second floor” contains 3 rooms: competent HIV care is integrated with women’s health [including sexual and reproductive health and rights (SRHR)] and mental health and addiction care; this is important as many women living with HIV do not see any other clinicians other than their HIV clinician. The “roof” contains peer support, leadership and capacity-building which are integral to the model. "The woman herself is the most essential component of the model and emphasizes the shared-care decision-making principle of person-centred care. The woman is often supported by a peer to enter the house and together the women are holding up the glowing piece about peer support. Importantly, the model is meant to be provided to all women, in all their diversity. Finally, women will be of different ages at different stages of their lives, so all components of women-centred HIV care are to be delivered across the life course.

The WCHC Model takes the form of a house (**Figure 2**) to signify safety and stability. This is in line with the Salamander Trust’s GVPS conclusions, which are also depicted in a house.^17^ The model has been created to be used by healthcare providers and women living with HIV, themselves (as self-management). Outside of the house is a path that indicates that women’s health and priorities will be assessed over their life course, acknowledging that women have different health and care priorities at different stages of life.

A “trauma- and violence-aware care” (TVAC) approach forms the foundation of the house, which acknowledges the high prevalence of violence and trauma experienced by women living with HIV, as well as the intersecting oppressions that many women experience. This approach aims to facilitate a sense of control and empowerment in a safe healthcare environment and to minimize re-traumatization by reframing power dynamics. Many care providers have heard of trauma-informed care (TIC)^48^ but while both incorporate anti-oppressive practices,^49^ TVAC expands the notions of TIC to consider the impacts of systemic violence and structural inequalities on a person’s life and health. The trauma and violence experts on our team emphasize that it may be better to use TVAC as *providers are not required to be experts in trauma and violence resolution*, which may be implied in the term “trauma-informed”. Rather, they need to be aware of the far-reaching impacts of various traumas on women’s lives and health, and act empathetically with that knowledge. TVAC embodies the basic principles of anti-oppression, anti-colonialism, and harm reduction, and aims to create safe, judgement-free spaces where healthcare delivery validates experiences and emphasizes resilience and strength. In Canada, where Indigenous women have been inordinately impacted by colonial violence, TVAC requires providers to be aware of the ongoing effects of settler colonialism on Indigenous peoples’ health, and practice cultural humility and safety at the individual level (e.g., avoiding paternalistic interactions), and advocate for safety at the structural level (e.g., the decolonization of our healthcare institutions).^50^ TVAC providers act with a sense of awareness regarding their role in provider-patient partnerships, making space for women’s voices and choices, thereby avoiding the perpetuation of institutionalized violence.

The “first floor” of the house affirms that the WCHC Model builds upon the already well-established person-centred care approach.^51^ Person-centred care, especially in the context of chronic diseases, evokes 4 central principles: 1) care provider empathy, commitment, and competence; 2) partnership in decision-making and shared responsibility between patients and providers; 3) prioritization of the patients’ illness experiences, expertise, and preferences; and, 4) a whole-person approach, inclusive of biological, psychological, and social context, such as culture, family, and socio-structural barriers to care.^51-53^ We use the term “person-centred care”, in lieu of “patient-centred care”, as it underlines the full spectrum of the lives of women living with HIV as whole persons rather than focusing on their ailments, or role as patients. Although ideal care of women living with HIV is shared care between the woman and her care provider(s), ultimately, the woman should feel able to claim her rights to autonomy over all decisions around her own life, health, and care. Person-centred care should be culturally- sensitive and -aware, as emphasized by many organizations, including the WHO, the Society of Obstetricians and Gynaecologists of Canada and the American College of Obstetrics and Gynecology. Person-centred care also emphasizes experiences of health across the life course, the relational aspect of care between individuals and providers over time, and explicitly considers multimorbidity—all aspects central to contemporary care of women living with HIV.^54^ In our WCHC Model, this component emphasizes attention to the social determinants of health and family. A person-centred care approach should attend to the factors that are often the most important to women’s lives; these often include the key social determinants (e.g., poverty, childcare demands, food, and housing needs) in shaping access to health and social care services, and family (e.g., children, parents and partners that live in Canada or abroad).

Next, the “second floor” contains 3 rooms that women can fluidly move between or access in combination at any given time: 1) competent HIV care, 2) competent women’s health care including SRHR, and 3) competent mental health and addiction care. Competent HIV care, in recent years, has been summarized by the components of the HIV care cascade including: diagnosis, linkage to care, retention in care, voluntary initiation of ART, and viral suppression.^55^ While clinicians should review the relevant importance of the HIV care cascade elements with their patients, it is ultimately up to each individual woman to decide what is best for her. Other indicators of competent HIV care include: screening and management of HIV-related comorbidities, minimizing antiretroviral toxicities and side effects, and providing high-quality, longitudinal patient education on issues ranging from HIV biology and transmission to HIV disclosure and legal considerations.

Fundamental to competent women’s health care is comprehensive SRHR care. This encompasses safe maternal care, and comprehensive gynaecologic care throughout the lifespan including contraception, pregnancy planning, appropriate screening and treatment of sexually transmitted infections (STI), cervical and breast cancers, treatment of breast and gynaecological diseases, and appropriate care during the transition into menopause and beyond. Supporting women in their social and health priorities, choices, and their rights to live healthy sexual lives is central to competent women’s health care (e.g., see resources such as Life and Love with HIV [www.lifeandlovewithhiv.ca]).^43^

Depression, post-traumatic stress disorder, and anxiety are common mental health experiences of women living with HIV, and thus competent mental health care is integral to their wellbeing. Providing competent mental health care involves screening for these common conditions. If the severity based on validated scoring is low to moderate, counselling can be provided through assessing the patient’s personal needs, desires, and experiences, and first-line pharmacotherapy could also be explored, if appropriate. For complex mental health conditions, such as multiple concurrent diagnoses, or those of high severity, having a care pathway to access a mental health professional locally is important. Assessment, support, and treatment of harmful substance use and addictions are an important element to be provided in a non-judgemental manner. The WHO has recently released a training manual for their 2013-2020 Comprehensive Mental Health Action Plan (https://www.who.int/mental_health/publications/action_plan/en/), which can be utilized by care providers.

The “roof” of the house highlights the importance of peer support, leadership, and capacity building as integral. The formative phase and stakeholder focus groups highlighted that peer support within HIV care is greatly desired and essential to combat HIV-associated isolation, stigma, and discrimination, as well as a much-valued source of education, social support, empowerment, and belonging.^16,24^ This is substantiated by other studies, which also suggest that social support fosters resilience and improves physical and mental health outcomes.^56,57^ Investing in social support, particularly peer support services, within HIV care may be critical to better engaging and supporting women.

In the WCHC Model, peer leadership and peer capacity building are distinct from peer support. While a growing number of organizations offer peer support groups and/or peer navigators, commitments to peer leadership and capacity building are limited. Peers in leadership positions play an essential role in advancing the health, wellbeing, and rights of women living with HIV, and involve positive benefits for peer leaders themselves.^57^ Peer leadership and capacity building entail that women living with HIV are not just *recipients* of peer support, but that care models prioritize and create opportunities for women to assume leadership roles in the design and delivery of care and to be hired into decision-making positions that affect their lives.^23,58^

Finally, an important aspect of the model is that the woman, herself, is larger and more important than the house and has the right to be fully informed and in control of her own care. The woman should have the support to be fully aware of her healthcare rights and responsibilities and to have the confidence to advocate for and manage them. Furthermore, there is another woman, a peer, holding up the roof’s circle piece of “peer support, leadership, and capacity building”, signifying that peers can be of support to each other to combat isolation. Also, the door is the same colour as the peer circle to signify that often, it is a peer who helps a woman enter into the house of safe care.

### Stakeholders’ Consultations and Considerations of Barriers

Stakeholder feedback suggested the following: to add addiction care to mental health care, as well as peer capacity building to peer support and leadership in the model; and to more deeply incorporate care over the life course. In addition to leading to revisions of the WCHC Model, the stakeholder consultations yielded consideration to who would be using the model and how. From baseline CHIWOS questionnaire data, it was identified that women in Canada receive HIV care from diverse care providers (59% from a specialist [e.g., an ID or internal medicine specialist], 24% from a combination of a specialist and a family physician, 9% from a family physician alone, 1% from a nurse/nurse practitioner, and 7% did not identify any type of HIV provider routinely involved in their care).^59^ The heterogeneity in HIV care delivery underscored the need for a flexible model that is adaptable to different care delivery settings depending on local resources, systems, realities, and potentially global circumstances. Other primary considerations include: 1) assessing the transferability of the model to other chronic illnesses and to care/service provider training; and 2) anticipating challenges in merging care that is currently generally siloed, such as SRHR and mental health.

### Other Consideration—Flexible Modes of Delivery

We determined that the WCHC Model must be flexible in its delivery through various modes of care delivery. These include delivery by a single provider such as a nurse, nurse practitioner or physician, a large clinic with multiple care providers, or multiple care clinics and organizations working together.^16,60^ The care could be delivered by a single primary care physician, nurse practitioner, or equivalent that is providing holistic person-centred care which integrates all aspects of the WCHC Model such that women only have to see one care provider for their visits (**Figure 3A**). In settings where the majority of women living with HIV are receiving HIV care from an ID specialist, the role of the specialist will need to be expanded to include the additional components of the model (**Figure 3B**) and/or increase the use of “shared care” models with community primary care providers (**Figure 3C**). In certain settings where the availability of services may be limited, adaptations to delivering the WCHC Model may be required. For example, in rural or remote regions of Canada, care may need to be provided by a single nurse or a team of nurses.

**Figure 3A.**
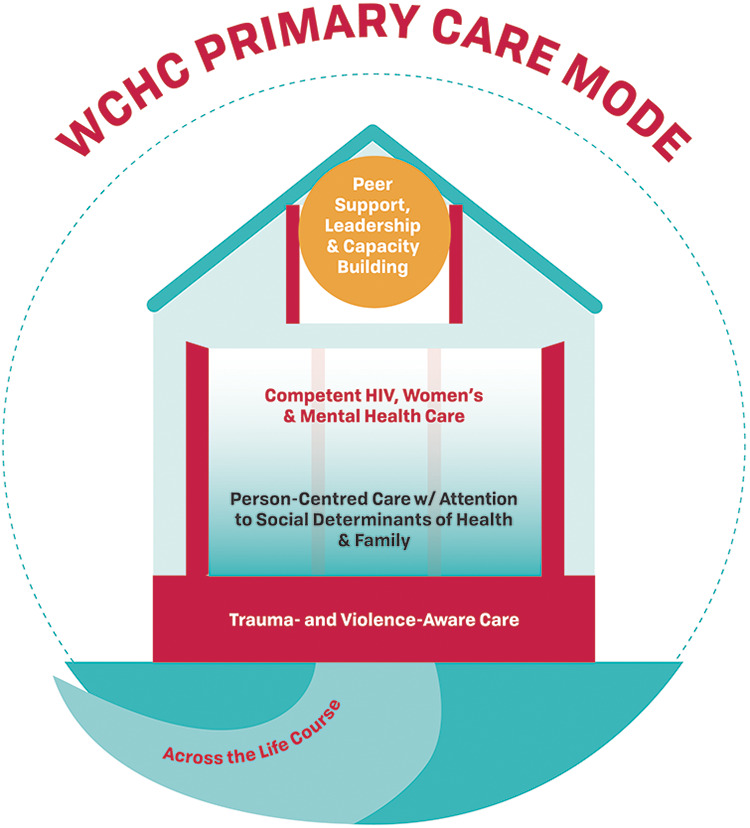
Primary care mode. One primary care clinician (physician, nurse, nurse practitioner, etc.) or clinic is providing all the care in a person-centred and holistic fashion.

**Figure 3B.**
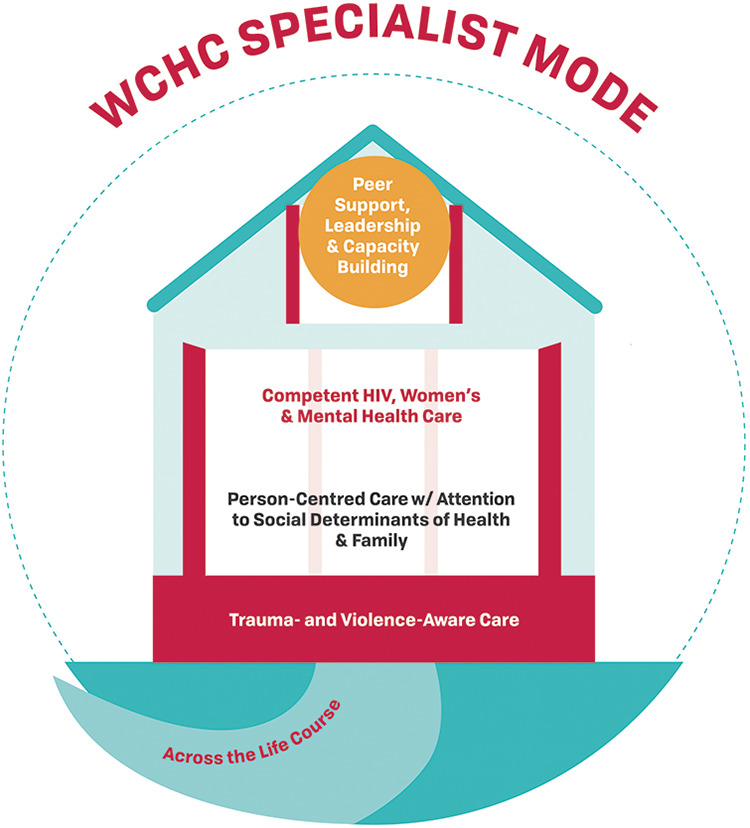
Specialist care mode. Infectious diseases or other specialist and/or clinic providing all WCHC components.

**Figure 3C.**
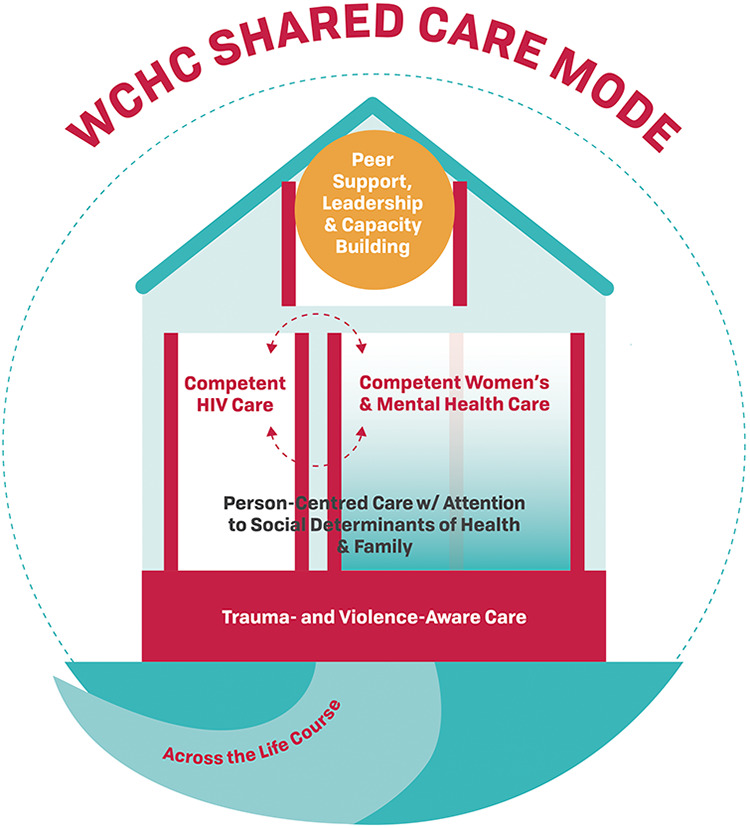
Shared care mode. Care is shared between a specialist and a primary care physician; both practice trauma- and violence- aware and person-centred care; however, the specialist minds the HIV care and the primary care provider cares for all other aspects with good communication between the 2.

A successful, existing example of the WCHC Model is the Oak Tree Clinic at the BC Women’s Hospital and Health Centre in Vancouver, BC (http://www.bcwomens.ca/our-services/specialized-services/hiv-care-for-women-families). At this interdisciplinary clinic, girls and women of all ages, receive fully integrated HIV, women’s health, and mental health care, along with social service advocacy and access to peer support and leadership opportunities in a single setting.

### Other Consideration—Usability in Various Settings

The model should be able to be delivered or received in an urban setting with numerous allied services or in rural/remote settings with limited facilities and resources. Rurality has been associated with poorer general and HIV-related health outcomes including mortality, late diagnosis of HIV, and slower uptake of novel ART agents.^61-63^ The setting is an important consideration for the WCHC Model as women with HIV living in rural and remote areas are those likely to receive the greatest benefit. In Canada, nearly 21% of the population lives in a rural region or small town.^62^ Although the data on HIV prevalence in rural Canada are limited, if the geographic distribution of women living with HIV is similar to the distribution in the general population, approximately 4,000 women living with HIV may be in rural and small communities and these women are an essential group to support.^62^ In BC, attrition along the HIV care cascade is highest in the northern region, which consists of predominantly rural and small communities.^64^ For women living with HIV in rural Canada, factors such as limited HIV expertise among care providers, confidentiality concerns, service inaccessibility due to geographic distance and a lack of comprehensive support programs act as further barriers to HIV care and overall good health.^65^ The issue of usability in rural and remote settings is heightened in Canada as its HIV epidemic disproportionately impacts Indigenous women and many Indigenous women live remotely in First Nations communities or Métis hamlets.^66^

Another setting to consider for the WCHC Model is for women who are incarcerated. Over one-third (37%) of women living with HIV in CHIWOS have experienced incarceration.^67^ In Canadian correctional settings, training and delivery of the model may be more suitable for healthcare providers working in federal facilities as women are imprisoned for 2 or more years in these facilities. However, provincial institutions are an important location to not miss the opportunity of connecting with women; more work is needed regarding person-centred HIV care in this setting.

### Other Consideration—Access to Health Care

Upon receiving feedback from international stakeholders, we realized that there is a minimum requirement in order to implement the WCHC Model: a community must have baseline access to health care for women living with HIV. A community must, at a minimum, have access to HIV testing and a nurse providing care, and have organizations or individuals cognizant of and actively addressing social barriers of access to care such as violence against women. Preferably, they would also have ART, contraception, STI testing and management access, and self-care interventions that support the autonomy and agency of women living with HIV.^68^ It is important to consider and address the fact that many communities may not have this basic access to care, which is a potential limitation of the WCHC Model.

## Discussion

After a ten-year endeavour, we have developed the WCHC Model. Our model is in line with the findings of the GVPS^17^ and the content of the WHO Consolidated guideline on SRHR of women living with HIV.^14,69^ The WCHC Model is a person-centred care model that underlines the importance of being attentive to and addressing violence and trauma and social determinants of health and family in the care for women living with HIV. In line with a global movement to prioritize integrated care,^70^ the WCHC Model integrates competent women’s health and mental health care with HIV care. While the integration of reproductive health and HIV care has been promoted for a decade,^71,72^ the integration of mental health care with HIV and women’s health care is novel. Integral to the model is the facilitation of peer connection and growth through peer support, leadership opportunities, and capacity building. Finally, our stakeholders made us aware of important implementation considerations, including that the care model must address the priorities of women living with HIV across their life course and should be flexible in its mode of delivery (i.e., can be delivered by a single provider, an interdisciplinary clinic, or multiple providers working collaboratively across sites). The WCHC Model must also be adaptable across diverse locales including urban, rural, and remote settings.

For delivery of the WCHC Model, it is recommended that healthcare providers and organizations liaise with CBOs, as important partners in the care of women living with HIV. HIV-specific CBOs are often the groups most connected to women living with HIV; thus, their partnership in this care model delivery is essential. CBO staff can not only offer tools, education, and opportunities to women living with HIV, they may also be the ones to engage and link women into medical care. Many CBOs already practice using anti-oppression and holistic person-centred care principles and therefore, they may easily adopt the WCHC Model. CBOs also offer a great opportunity to support women to connect with peers and to train women on how to practice self-management and health self-advocacy. Working with peer navigators to assist women in building knowledge and skills to navigate the system and advocate for their own care is becoming increasingly common at CBOs. This also contributes to the empowerment of women through leadership roles and capacity building.

There are limitations to our model development. The methods and synthesis of our findings to develop our care model, while contributed to by over 100 collaborators including women living with HIV, are partly subjective. We did obtain extensive stakeholders’ feedback and input in efforts to increase the model’s adaptability. Other limitations include a lack of expansion on cultural and gender competence; however, we believe that such competencies are a part of person-centred care. As our model is based firmly on inclusivity, it is important to recognize that some cultural groups may not relate to a Western/allopathic approach to health care, and that our care model may need to be expanded to better address the health and social concerns of specific populations, such as Indigenous women, trans women, and women of diverse ethnicities and identities. Additionally, the symbolism of our model in the shape of a house—that is secure, safe and with multiple rooms—may not resonate with all women living with HIV. Finally, our model is contingent on resource availability for care delivery. However, all components of the model do not need to be in place at once; new elements can be added as resources become available. This limitation is also an opportunity to channel advocacy efforts for comprehensive care and access for all women living with HIV.

## Conclusions

Ultimately, the WCHC Model is a result of collective research and discussions that concluded in new ways to address the health and wellbeing priorities of women living with HIV across their life course. Our findings echo the experiences of women living with HIV in various countries with a range of available resources and health care contexts; studies from both high- and low-resource settings have demonstrated the similarities of HIV issues across continents, such as the feminization of HIV, HIV as a chronic disease when there is access to HIV testing, care and ART, HIV-related stigma, and complex social positioning of women within society.^17^ These global similarities suggest that the WCHC Model (or components of it), may be applicable to diverse settings within and beyond Canada. The model as an intervention must be tailored to local contexts, values and preferences, in consultation with women living with HIV, as suggested in the WHO guideline.^14^ The next step is to operationalize this model and to do so we are developing 2 toolkits.^73^ The toolkits are designed to provide clear directions to healthcare providers on how to deliver WCHC and to women living with HIV on how to best self-manage their health and on how to be a health self-advocate. Finally, the WCHC Model and toolkits could be used as advocacy tools to promote and protect human rights and gender equality.^14,58,74^ The consensus among women living with HIV in the GVPS^17^ is clear: “all women—including those living with HIV—need humane, holistic services that take into account the different stages of a woman’s life”.^75^(p. 247)
